# Development and validation of a clinical prediction model for blastocyst formation during IVF/ICSI-ET

**DOI:** 10.3389/fendo.2024.1432943

**Published:** 2024-12-09

**Authors:** Xingnan Liu, Jingyun Zhao, Yi Zhang, Zhaoyan Nie, Qiaoxia Li, Lina Guo, Chunhui Fan, Jianfeng Zhang, Na Zhang

**Affiliations:** ^1^ Department of Reproductive Medicine, The Fourth Hospital of Hebei Medical University, Shijiazhuang, China; ^2^ Department of Orthopaedic Surgery, Third Hospital of Hebei Medical University, Shijiazhuang, China

**Keywords:** *in vitro* fertilization, clinical prediction model, blastocyst formation, cleavage embryos, nomogram

## Abstract

**Purpose:**

This study aims to create and validate a clinical model that predict the probability of blastocyst formation in IVF/ICSI-ET cycles.

**Methods:**

This study employed a retrospective methodology, gathering data from 4961 cleavage-stage embryos that cultured in the reproductive center’s of the Fourth Hospital of Hebei Medical University between June 2020 and March 2024. 3472 were in the training set and 1489 were in the validation set when it was randomly split into the training set and validation set in a 7:3 ratio. The study employed both univariate and multivariate logistic regression analysis to determine the factors those influence in the process of blastocyst formation. Based on the multiple regression model, a predictive model of blastocyst formation during IVF was created. The calibration and decision curves were used to assess the effectiveness and therapeutic usefulness of this model.

**Results:**

The following factors independently predicted the probability of blastocyst formation: the method of insemination, number of oocytes retrieved, pronuclear morphological score, the number of cleavage ball, cleavage embryo symmetry, fragmentation rate and morphological score and basal P levels of female. The receiver operating characteristic curve’s area under the curve (AUC) in the training set is 0.742 (95% CI: 0.724,0.759), while the validation set’s AUC is 0.729 (95% CI: 0.703,0.755), indicating a rather high clinical prediction capacity.

**Conclusion:**

Our generated nomogram has the ability to forecast the probability of blastocyst formation in IVF, hence can assist clinical staff in making informed decisions.

## Introduction

As assisted reproductive technology continues to progress, more theories favor the ultimate goal of having healthy term single births. Therefore, transplanting the minimum number of embryos has been selected for most reproductive centers without affecting pregnancy rates and live birth rates. Therefore, it is particularly important to screen for embryos. Clinically, embryos are evaluated by embryologists and transferred at cleavage stage on day 3 (D3) or blastocyst stage on day 5 (D5) (1). The blastocyst-stage embryo is the end of *in vitro* embryonic culture, typically formed at five to six days after oocyte fertilization. Advances in embryo culture systems promote the currency of moving toward blastocyst transfer (2). Blastocyst transfer allows for self-selection of cleavage embryos with high developmental potential, thus maximizing pregnancy rates and live birth rates in fresh cycles (3). Other advantages of blastocyst transfer include: Better temporal synchronization between embryonic and endometrial development at the time of embryo transfer; Reduced multiple pregnancies through transferring fewer embryos; Higher implantation rates and so on (4, 5).

However, reports indicate that blastocyst formation rates range from 31% to 73%, and the lower formation rate of blastocysts allows for an increased risk of fresh-cycle transplant cancellation (6).Young patients with good ovarian response may benefit from blastocyst transplantation, but for patients with poor ovarian response or advanced age, blastocyst transplantation may increase the risk of cycle cancellation, which brings to them a heavy psychological and economic burden. Therefore, it is crucial to predict the probability of blastocyst formation based on the cleavage embryo characteristics and the hormone levels of both infertile couples.

There is a large amount of literature to explore the influence of *in vitro* fertilization laboratory culture conditions on blastocyst formation, and the predictive value of prokaryotic cells and cleavage embryo morphology on blastocyst formation (7). However, there is no relatively comprehensive clinical application model for accurate prediction. Therefore, the goal of this paper is to establish a clinical prediction model to predict the probability of blastula formation based on the parameters of female age, hormone level, controlled ovulation stimulation characteristics, sperm parameters, and cleavage-stage embryos, in order to provide tools for clinical decision-making.

## Materials and methods

### Sample selection

The retrospective analysis was performed on patients receiving IVF/ICSI in the Reproductive Medicine Department of the Fourth Hospital of Hebei Medical University from June 2020 to March 2024.This study was approved by the Ethics Committee of the Reproductive Department of the Fourth Hospital of Hebei Medical University, and all patients gave informed consent. The inclusion criteria: 1. The women were less than or equal to 40 years old; 2. Couples performed IVF/ICSI-ET cycles in our reproductive department; 3. There were still remaining embryos in the frozen embryo transfer cycle that can be blastocyst culture or fresh cycle blastocyst transfer. The following were the exclusion criteria: 1. The women were older than 40 years old; 2. No embryos available for continued blastocyst culture; 3. Preimplantation genetic testing (PGT/PGD); 4. Other diseases that may affect pregnancy outcome, such as uterine malformation, history of adverse pregnancy, endometriosis, diabetes, hyperprolactinemia, abnormal thyroid function, history of pelvic tuberculosis. Finally, 4691 continued cleavage-stage embryos were included in this study.

### Controlled ovulation stimulation protocol

The individualized ovarian stimulation program is designed based on the female’s age, basic hormone levels, and ovarian reserve function. This program involves monitoring follicle growth, through transvaginal ultrasound, and adjustments are made according to the size of the dominant follicle and hormone levels. When the appropriate criteria are met, the patient is injected with human chorionic gonadotropin (hCG) or gonadotropin releasing hormone (GnRH-a), followed by a vaginal vault puncture to retrieve the oocytes. This procedure is conducted 36 hours after the trigger day.

### Semen treatment

On the day of oocyte retrieval, the male partner provided sperm by masturbating. After that, the sperm underwent density gradient centrifugation. Routine *in vitro* fertilization and intracytoplasmic sperm injection were performed in the center (8).

### Embryo culture and assessment

#### Pronuclear observation and morphological scores

The fertilized oocytes were placed in a CO_2_ incubator for embryo culture. Egg fertilization was observed after 16-18 hours of fertilization. If the pronuclear appeared in the cytoplasm of the egg, it was fertilization. The PN=2 was defined as normal fertilization, and the rest was abnormal fertilization. Pronuclear morphological score criteria was performed according to the Z-scoring method (9). According to the size, number and arrangement of prokarytes and nucleoli, 2PN pronuclei were divided into Z1-Z4 grades. Two pronuclear were equal in size, close to each other, nucleoli size and number of equal, the number was three to seven, and the polar symmetry arrangement was called Z1 level; There was no polar symmetry arrangement was called Z2 level; The nucleoli were different in size and number and had no polar symmetry arrangement was called Z3 level; The two pronuclear were different in size or not close to each other, and the nucleolar arrangement was called the Z4 level.

#### Morphological score of cleavage embryos

According to the Peter standard (10), cleavage embryos were observed at 36 hours after fertilization, and the embryonic cell number greater than or equal to two was defined as cleavage. The blastomeres were uniform, with regular morphology, complete zona pellucida, uniform and clear cytoplasm, no granular phenomenon, and the embryo with no or little debris (<5%) was defined as grade I. The blastomeres were slightly uneven, slightly irregular morphology, the zona pellucida was complete, the cytoplasmic particle phenomenon, the debris 6% -20% was defined as gradeII; The blastomeres were significantly heterogeneous, with irregular morphology, large cytoplasmic particles and a 21%-50% fragments was defined as grade III. The blastomeres were severely inhomogeneous, and the cytoplasmic particles were severe, with debris> 50% was defined as grade IV.

Except for the embryos transferred, all available embryos were selected for blastocyst culture.

### Data collection

Age, Body Mass Index (BMI) were recorded as the clinical parameters for infertile couples. The levels of basal estrogen, luteinizing hormone (LH), and follicle stimulating hormone (FSH) in females, as well as estrogen, progesterone, and LH levels on the trigger day, were also measured. The mature follicular rate was assessed, along with semen parameters on the day of oocyte retrieval, including semen volume, percentage of morphologically abnormal sperm, sperm vitality, sperm concentration, and PR%. In addition, embryonic parameters for the various stages were included, such as pronuclear morphological score, number of cleavage ball, symmetry, fragmentation rate and morphological score of cleavage embryo. Successful blastocyst formation was considered as a positive event.

### Statistical analysis

According to the random sampling technology, all of the cleavage-stage embryos were divided into training set and validation set in a 7:3 ratio. The continuous variables were represented as the mean ± standard deviation (SD), while non-normally distributed data were presented as the median (interquartile range). To compare variables between groups, Student’s t-tests (for normally distributed data) or the Mann–Whitney U-test (for non-normally distributed data) were employed. Categorical variables were expressed as percentages, and the chi-squared test was used for statistical comparison. These data were analyzed using SPSS 23.0.

Univariate logistic regression was used to identify predictive factors associated with blastocyst formation. The variables with P<0.05 were entered into the next multifactor analysis. To reduce overfit bias, internal validation was performed using bootstrap resampling. Bootstrapping repeated the process of drawing samples with replacement from the original dataset 500 times. The closer the original and corrected statistics, the better the fit of the regression model (11). The area under the ROC curve was used to assess the accuracy of the nomogram. Additionally, a decision curve analysis was performed to determine the clinical utility of this model. The statistical analysis mentioned above was conducted using IBM SPSS Statistics for Windows (version 23.0) and R (version 4.3.1). P value less than 0.05 was considered statistically significant.

## Results

### Baseline characteristics

A total of 4691 cleavage embryos were included in this paper, of which 3139 embryos successfully formed blastocysts, accounting for about 63.2%. The training set and validation set were split according to the ratio of 7:3. According to the baseline table in **Table 1**, there was no statistical difference between the basic characteristics of the two groups (*P*<0.05).

**Table 1:** Basic characteristics of the training and validation sets.

Characteristics	Validation set (n=1489)	Training set (n=3472)	P-Value
Female age	31.0 [28.0;33.0]	31.0 [29.0;34.0]	0.234
Male age	31.0 [29.0;34.0]	31.0 [29.0;34.0]	0.739
Female BMI	23.4 [21.0;26.8]	23.4 [20.9;26.6]	0.368
Male BMI	25.9 [23.1;28.4]	25.6 [23.1;28.3]	0.512
Basal FSH of female	6.27 [5.36;7.63]	6.24 [5.25;7.54]	0.088
Basal E_2_ of female	41.7 [31.1;55.8]	41.5 [31.3;54.4]	0.47
Basal P of female	0.27 [0.18;0.40]	0.25 [0.17;0.39]	0.095
Basal PRL of female	15.1 [11.3;20.4]	15.1 [11.2;20.6]	0.909
Basal LH of female	5.27 [3.72;7.52]	5.33 [3.71;7.76]	0.718
The method of fertilization			0.305
IVF	1293 (86.8%)	3053 (87.9%)	
ICSI	196 (13.2%)	419 (12.1%)	
Starting dosage of Gn used	225 [150;250]	225 [150;250]	0.682
Total dosage of Gn used	2425 [1775;3150]	2450 [1775;3150]	0.805
Total number of days of Gn used	11.0 [9.00;13.0]	11.0 [9.00;13.0]	0.367
Controlled ovarian stimulation protocol			0.224
GnRH-ant protocol	816 (54.8%)	1833 (52.8%)	
GnRH-a protocol	592 (39.8%)	1406 (40.5%)	
The other	81 (5.4%)	233 (6.7%)	
Controlled ovarian stimulation drugs			0.557
HMG	339 (22.8%)	899 (25.9%)	
rFSH-α	667 (44.8%)	1444 (41.6%)	
rFSH-β	483 (32.4%)	1129 (32.5%)	
number of oocytes retrieved	17.0 [11.0;22.0]	17.0 [11.0;21.0]	0.571
E2.on.the.day.of.HCG.trigger	3000 [2147;5257]	3000 [2186;5140]	0.894
LH.on.the.day.of.HCG.trigger	1.15 [0.74;2.05]	1.18 [0.73;1.95]	0.694
P.on.the.day.of.HCG.trigger	0.82 [0.53;1.26]	0.83 [0.51;1.26]	0.729
Semen.volume	2.50 [2.00;3.00]	2.50 [2.00;3.00]	0.377
Sperm.concentration	40.0 [30.0;50.0]	40.0 [30.0;50.0]	0.660
PR%	35.0 [30.0;40.0]	35.0 [30.0;40.0]	0.839
Normal.morphology.rate.of.the.semen	3.00 [2.00;4.00]	3.00 [2.00;4.00]	0.010
Sperm.vitality	40.0 [35.0;45.0]	40.0 [35.0;45.0]	0.839
Pronuclear morphological score			0.844
Z1	31 (2.08%)	74 (2.13%)	
Z2	175 (11.8%)	399 (11.5%)	
Z3	1147 (77.0%)	2707 (78.0%)	
Z4	136 (9.13%)	292 (8.41%)	
Number of cleavage ball	8.00 [6.00;8.00]	8.00 [7.00;8.00]	0.026
Cleavage embryo morphological score			0.517
I	481 (32.3%)	1118 (32.2%)	
II	669 (44.9%)	1614 (46.5%)	
III	339 (22.8%)	738 (21.3%)	
IV	0 (0.00%)	2 (0.06%)	
Cleavage embryo fragmentation rate	8.00 [0.00;20.0]	8.00 [0.00;20.0]	0.972
Cleavage embryo symmetry			0.487
Yes	1116 (74.9%)	2636 (75.9%)	
No	373 (25.1%)	836 (24.1%)	

Continuous variables are shown as the median (interquartile range) or mean ± standard deviation. Categorical variables are presented as percent.Student's t-tests (for normally distributed data) or the Mann–Whitney U-test (for non-normally distributed data) were employed. Categorical variables were expressed as percentages, and the chi-squared test was used for statistical comparison.

BMI, body mass index; FSH, follicle-stimulating hormone; LH, luteinizing hormone; P, progesterone; E2, estradiol; *Training set vs. validation set: P < 0.05.

### Logistic regression analysis

**Table 2** showed the univariate logistic regression analysis of the factors influencing blastocyst formation, and the results showed that the age of the infertile couples and the levels of basal E2, P, and PRL in females were statistically different between the two groups (*P*<0.05). The controlled ovulatory process can also affect blastocyst formation, such as the starting dose and total dose of Gn used and increased estrogen levels on the trigger day can reduce the probability of blastocyst formation (*P*<0.05). According to **Table 2**, compared with ICSI, traditional fertilization was more conducive to blastocyst embryos formation, and the probability of blastocyst formation increased with the number of oocytes retrieved (*P*<0.05). In addition, the quality of the embryos that we were most concerned about also affected blastocyst development. And the rate of blastocyst formation decreased with the decline of pronuclear morphological score and cleavage embryo morphological score. The increase of fragmentation rate and inhomogeneity of cleavage stage embryos also affected blastocyst formation (*P*<0.05).

**Table 2:** Univariate analysis in the training set.

Variables	OR	CI	*P*-Value
Cleavage embryo symmetry	0.369	(0.314-0.432)	<0.001
Cleavage embryo fragmentation rate	0.953	(0.947-0.958)	<0.001
Cleavage embryo morphological score	0.417	(0.375-0.462)	<0.001
Number of cleavage ball	1.376	(1.319-1.436)	<0.001
Pronuclear morphological score	0.807	(0.704-0.922)	0.002
E2.on.the.day.of.HCG.trigger	1	(1-1)	0.002
Number of oocytes retrieved	1.016	(1.007-1.025)	<0.001
Total.dosage.of.Gn.used	1	(1-1)	0.011
Starting.dosage.of.Gn.used	0.999	(0.998-1)	0.012
The method of fertilization	0.531	(0.433-0.653)	<0.001
Basal.PRL.of.Female	0.998	(0.997-1)	0.046
Basal.P.of.Female	0.947	(0.902-0.986)	0.014
Basal.E_2_of.Female	1	(1-1.001)	0.039
Male.Age	0.978	(0.963-0.993)	0.005
Female.Age	0.979	(0.963-0.995)	0.012

OR, odds ratio; CI, confidence interval.

The factors in **Table 2** were further included in the multivariate logistic regression analysis, and we obtained **Table 3**, which showed that the independent predictors of blastocyst formation were the method of fertilization (OR: 0.661, 95%CI: 0.528,0.829, *P*<0.001), the number of oocytes retrieved (OR: 1.011, 95%CI: 1.001,1.021, *P*=0.021), pronuclear morphological score (OR: 0.812, 95%CI: 0.699,0.941, *P*=0.006), number of cleavage ball (OR: 1.325, 95%CI: 1.268,1.386, *P*<0.001), cleavage embryo morphological score (OR: 0.713, 95%CI: 0.598,0.85, *P*<0.001), cleavage embryo fragmentation rate (OR: 0.975, 95%CI: 0.966,0.985, *P*<0.001), cleavage embryo symmetry (OR: 0.475, 95%CI: 0.394,0.572, *P*<0.001) and the basal P level of females (OR: 0.952, 95%CI: 0.906,0.993, *P*<0.001).

**Table 3:** Multivariate logistic regression model in the training set.

Variables	OR	CI	P-Value
The method of fertilization	0.661	(0.528-0.829)	<0.001
Number of oocytes retrieved	1.011	(1.002-1.021)	0.021
Pronuclear morphological score	0.812	(0.699-0.941)	0.006
Number of cleavage ball	1.325	(1.086-1.282)	<0.001
Cleavage embryo morphological score	0.713	(0.598-0.85)	<0.001
Cleavage embryo fragmentation rate	0.975	(0.966-0.985)	<0.001
Cleavage embryo symmetry	0.475	(0.394-0.572)	<0.001
Basal.P.of.Female	0.952	(0.906-0.993)	0.033

### Development and validation of the clinical prediction model

**Figure 1:**
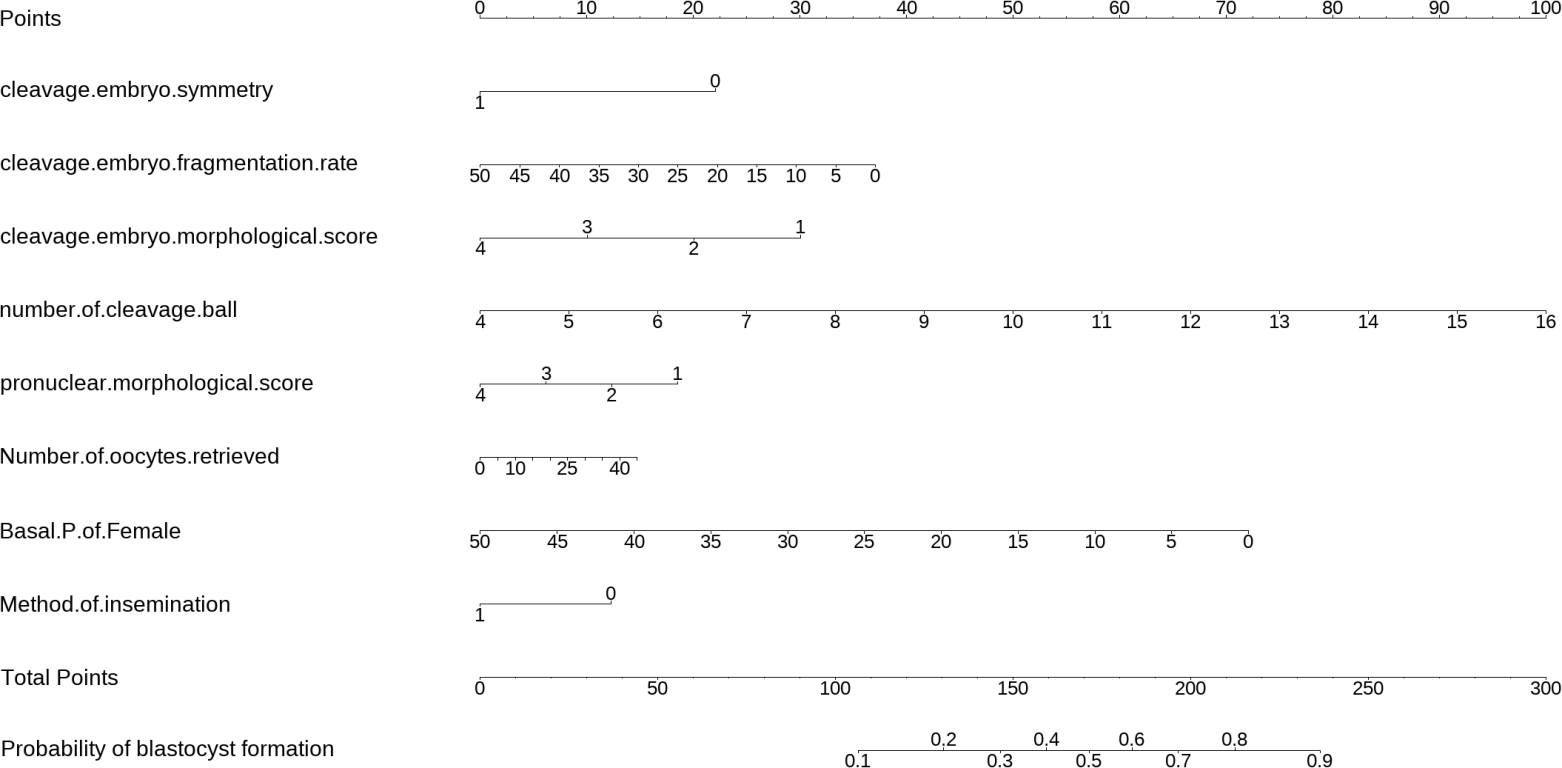
The nomogram to predict the probability blastocyst formation in routine *in-vitro* fertilization (IVF) cycles. The nomogram can be applied by following procedures: draw a line perpendicular from the corresponding axis of each risk factor until it reaches the top line labeled “Points”; sum up the points for all risk factors and recorded as the total score; and draw a line descending from the axis labeled “Total points” until it intercepts the lower line to determine the probability of blastocyst formation. The optimal threshold point was calculated using receiver operating characteristic (ROC) curve.

**Figure 2:**
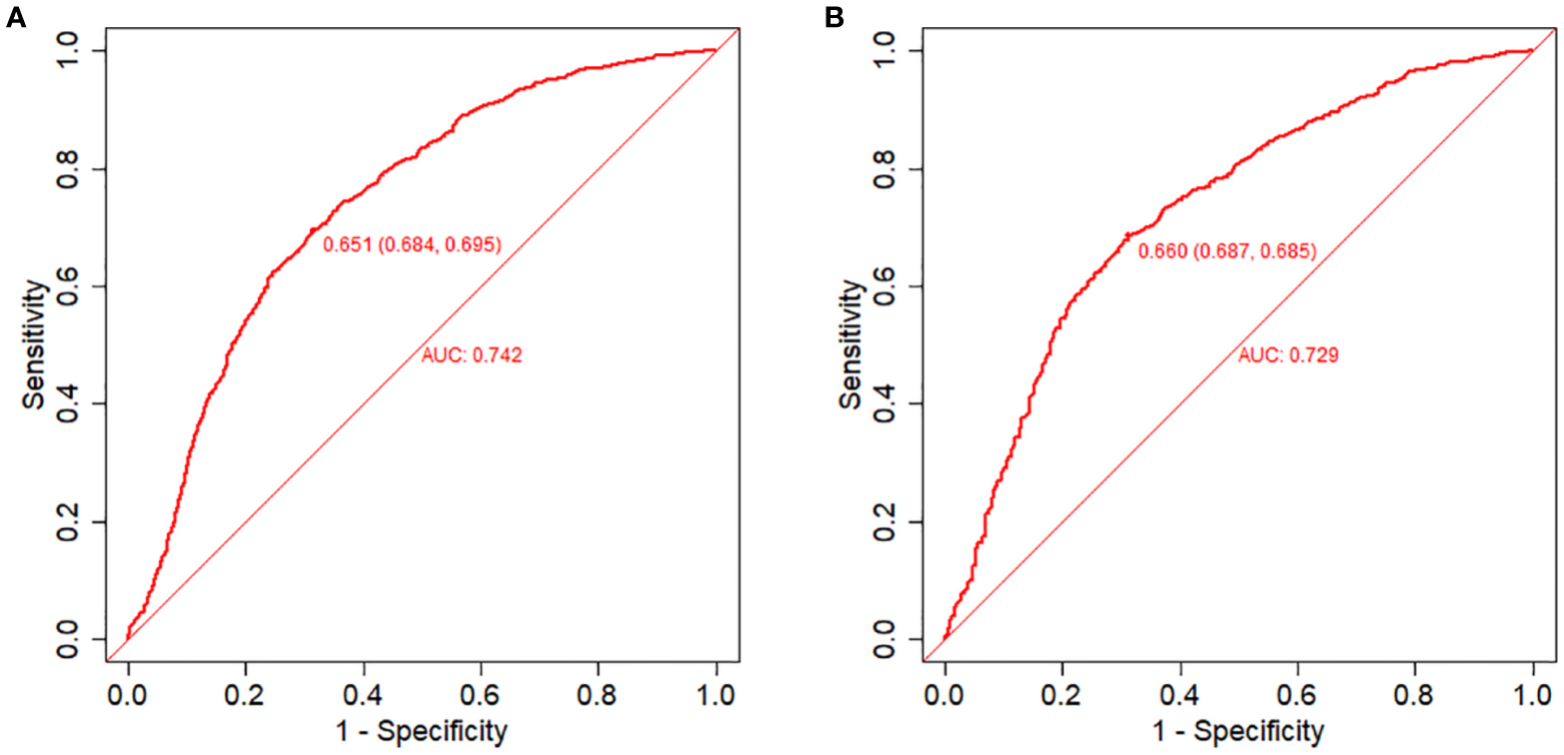
Receiver operating characteristic (ROC) curves and calibration plots of the training and validation sets. **(A)** Area under the ROC curve (AUC) of the training set is 0.742 (95% CI: 0.724,0.759). **(B)** AUC of the validation set is 0.729 (95% CI: 0.703,0.755).

**Figure 3:**
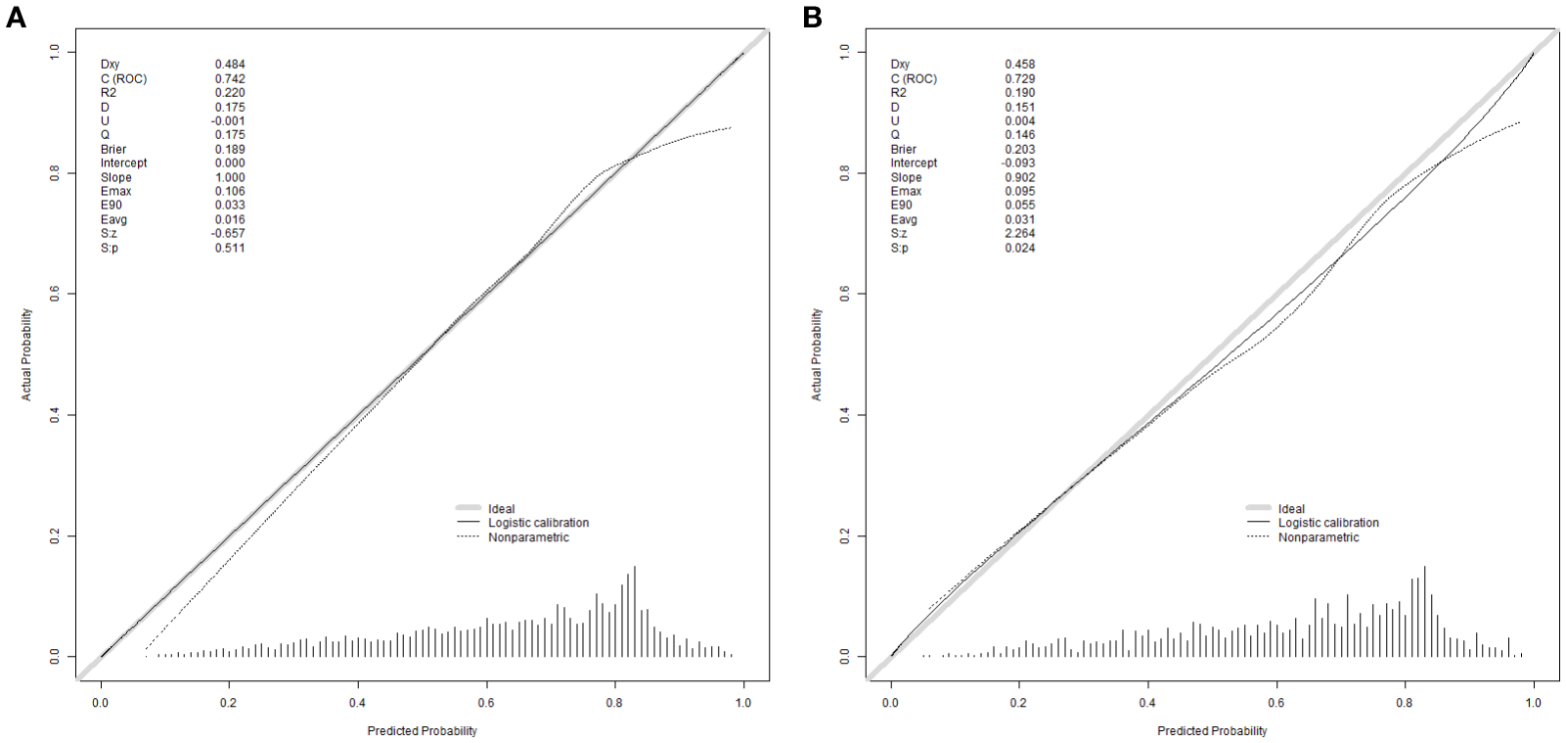
Calibration curves were used to evaluate the calibration of the model. The horizontal axis is the predicted probability provided by this model, and the vertical axis is the observed incidence of blastocyst formation. The ideal line with 45° slope represents a perfect prediction (the predicted probability equals the observed probability). The lower the Brier score for a set of predictions, the better the prediction calibration. When the slope was closer to 1.00, the prediction model had better calibration power. **(A)** Calibration curve for training set (Brier = 0.189, Slope = 1.000). **(B)** Calibration curve for validation set (Brier = 0.203, Slope = 0.902).

**Figure 4:**
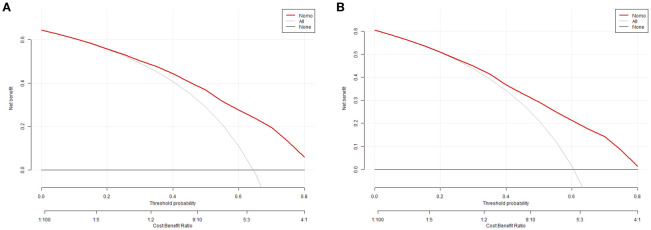
the decision curve analysis of the training set **(A)** and validation set **(B)**.

The equations were constructed using regression coefficients to determine the probability of blastocyst formation (P) = 0.117 -0.414* The method of fertilization + 0.011 *The number of oocytes retrieved -0.209 *Pronuclear morphological score +0.282* The number of cleavage ball- 0.339 * Cleavage embryo morphological score -0.025 * Cleavage embryo fragmentation rate - 0.745 * Cleavage embryo symmetry - 0.049 *The basal P level of females. To predict the probability of blastocyst formation in conventional IVF, we developed a nomogram (**Figure 1**), that includes of the above independent predictors. The area under the receiver operating characteristic curve (AUC) for the training set (**Figure 2A**) is 0.742 (95% CI: 0.724,0.759), indicating good clinical predictive ability. Similarly, the validation set (**Figure 2B**) has an AUC of 0.729 (95% CI: 0.703,0.755). The calibration curves for the training set (**Figure 3A**) and validation set (**Figure 3B**) have slopes of 1.000 and 0.00, respectively, indicating good calibration ability. Furthermore, the decision curve analysis of the training set (**Figure 4A**) and validation set(**Figure 4B**) demonstrates that the prediction model has high net income and clinical application value, as it is positioned higher on the decision curve.

## Discussion

Since the development of *in vitro* fertilization-embryo transfer technology, embryo morphological analysis has been used as an important tool to assess embryo development, and the selection of high-quality embryos by non-invasive methods is crucial (11, 12). At the same time, selecting a high-quality blastocyst to achieve a singleton pregnancy is the ultimate goal of IVF-ET. Continuing the cultivation of cleavage-stage embryos to the blastocyst stage is considered an effective method for the screening of single blastocysts with high implantation potential for embryo transfer to reduce the occurrence of multiple pregnancies (13). The basic principle of blastocyst culture is to improve the synchronization of endometrial and embryonic development, and to select blastocysts with higher developmental potential, to some extent eliminating embryos with low developmental potential or genetic defects. Thus, the available blastocysts are selected for transplantation, and ultimately further improve the embryo implantation rate (14). So increasing the time for embryos to grow *in vitro* provides a good opportunity for embryo self-selection. The culture of blastocyst has been restricted by many factors, among which the age of the woman, the number of embryonic blastomeres, fragmentation rate, the uniformity of blastomeres, cytoplasmic mass and embryo level all have an influence on the formation of blastocyst.

In terms of female fertility levels, their fertility declines with age, which may be due to the reduced number of female oocytes and the inability of the embryos to develop into blastocysts (15). It has been shown that with aging, the number of embryonic chromosome abnormalities increases and the possibility of blastocyst formation declines (16). Therefore, increasing the total number of oocytes retrieved improves the blastocyst formation probability, which is consistent with our findings.

The number of cleavage ball is a key indicator of embryonic developmental potential because it can directly reflect the developmental progression of the embryo (17). Studies have found that the number of cleavage ball is less than five shows a small developmental potential. This phenomenon may be mainly caused by oocyte factors, but the cause of retardation is still unclear (18). Another study found that compared with faster division embryos, selection with six to nine cells cleavage embryos shows more advantage (19). This is consistent with our results.

It is well known that the increase in embryo fragmentation rate is directly related to the decreased embryo implantation rate and pregnancy rates in IVF (20). For high-quality blastocyst formation, cleavage embryo fragmentation rate remains one of the influencing factors. A high degree of fragmentation rate causes the loss of the cytoplasm, organelles such as mitochondria. And will further induce the apoptosis of the cycle blastomeres, which leads to embryonic development arrest (21). Further affecting the potential of later embryo development to the blastocyst. Studies have found that the rate of blastocyst formation decreases with the increase of cleavage embryo fragmentation rate (22). Thus, the morphological score of cleavage-stage embryos receives the effect of the fragmentation rate, thereby affecting blastocyst formation.

For the current study, it is unclear whether ICSI techniques have detrimental effects on embryonic development and blastocyst formation. However, some studies found that the ratio of excess embryos obtained by the IVF cycle formed blastocysts was significantly higher than the ICSI cycle, and that the developmental potential of the remaining cleavage stage embryos in the IVF group was higher than that of the ICSI group (23). This is consistent with the results of the present study. The possible mechanism is that the egg granulosa cells prefer the sperm during IVF insemination, and finally the sperm DNA integrity is high. ICSI insemination selects better morphology of sperm for injection, unable to identify the internal structure of sperm (24). ICSI fertilized embryos had poor semen quality, which may be one of the important factors affecting blastocyst formation.

## Conclusions

We discovered that factors such as the method of insemination, number of oocytes retrieved, pronuclear morphological score, the number of cleavage ball, cleavage embryo symmetry, fragmentation rate and morphological score and basal P levels of female independently predicted the likelihood of blastocyst formation. Our retrospective study has developed a well-calibrated model that accurately predicts the probability of blastocyst formation IVF treatment. This model carries significant clinical implications. This can provide a better reference for patients with fewer oocytes to undergo blastocyst culture.

## Data Availability

The raw data supporting the conclusions of this article will be made available by the authors, without undue reservation.
